# Fleas and Babies

**Published:** 1908-11-28

**Authors:** 


### Diseases of Children.

### FLEAS AND BABIES.

One of the minor annoyances of life is the peculiarly irritating result of the bite of the insect known in technical language as pulex irritans and in common parlance as the flea. No one seems to have a good word for this obnoxious little insect, and, indeed, it is seriously regarded as a most dangerous parasite, with no redeeming virtues, and a possible source of the spread of epidemic disease such as plague.

Even the adult, who can bring skill and intelligence to the pursuit and capture of his foe, suffers discomfort and perhaps sleepless nights in consequence of the irritation of the bites. Some individuals are much more susceptible than others, not only to the attacks of the insect but also to the subsequent reaction. One may be rarely bitten or, if bitten, show nothing but a minute puncture mark, while another is rabidly devoured and gives evidence thereof in large red bumps, scattered generally all over the body or following an irregular line as the flea progresses in its gustatory course along a limb or over the trunk.

Babies suffer intolerably from the pest, unless they are carefully watched and freed from these parasites. It is quite common in hospital outpatient departments to see babies covered from head to foot with recent and old bites, and it is rare to find such a baby healthy or well nourished. Apart from the irritation of the bites, they may be taken as direct proof of the carelessness and neglect of the mother, for no mother with the least affection for her child, unless grossly incompetent, would suffer it to undergo the tortures of irritation to which such a mass of bites subject it. Fortunately, after a certain period the irritability seems to decrease very considerably. A baby unused to fleas will generally at once give evidence of the attack by wriggling movements, scratching, or fretful cries. One who has been freely bitten will pay not the least attention, though several of the insects may be gorging simultaneously.

Restless nights are a common sequel. Many a baby is partially or entirely weaned because the crying and disturbed sleep are put down to indigestion or an insufficient supply of breast milk. As the baby ages and develops its nails and a vigour sufficiently great to indulge in scratching, a new danger is incurred. The tops of the bites are exposed and bleed, raw surfaces and linear scratches are formed, with the result that secondary infection is by no means uncommon, ending in various pustular rashes, sometimes ecthymatous, and in rare instances in a local gangrenous dermatitis.

There are several remedies for the prevention or destruction of fleas but not all are suitable for small children. A medical student with a drop bottle of chloroform will dash on a few drops where he feels the bite, and then rapidly exposing the part capture the stupefied insect. Carbon disulphide can be used in a similar way, but it is very inflammable and soon develops an unpleasant smell. Formalin, 40 per cent., applied to the bedclothes, pyjamas, etc., is irritating to the respiratory organs and very unsuitable for infants. Essence of thyme and powdered cloves are fairly efficacious when sprinkled over the clothes and bed-linen. Carbolic lotions are useful, if applied generally, though not to be recommended for infants because of their habit of putting things in the mouth. Tincture of pyrethrum or the oils of musk, cinnamon, cloves, citronella, lavender, and eucalyptus applied externally are useful and not unpleasant. A very simple remedy is to wrap up a few pledgets of cotton wool, sprinkled with a 50 per cent. solution of Jeyes' fluid, with the night garments during the day time, for it is said to keep away the fleas at night. Keating's powder is efficacious but is unsuitable, for the child may get some into its mouth.

Of course, the simplest treatment of all is to catch the fleas, and this should invariably be the first advice given. Next, have all the clothes, bed and body linen of infested cases thoroughly stoved or boiled to destroy the eggs. The nursery should contain no carpet and the floor be covered with linoleum, daily washed with some harmless disinfectant such as Izal. Where the whole house is infested the rooms should undergo thorough cleaning, fumigation, white-washing, papering, and painting. In fact, all rooms, furniture, clothing, etc., require as careful disinfection as in the case of infectious fevers. Bugs are even more difficult to get rid of. Their effects on the unfortunate baby are worse than those of fleas. They inhabit the woodwork of beds and rooms, and the cracks of the plaster. Consequently, it will be necessary to have all the old lath and plaster of the ceilings and walls of infested rooms removed and destroyed. Midges, mosquitoes, and other insects of a similar nature bite and cause a local itching swelling, very like urticaria, occasionally like the nodules of erythema nodosum. The effects vary greatly in different children. Sometimes the swelling is enormous and simulates angioneurotic œdema. Flies, too, may cause serious and even fatal injury. The ordinary bite is merely slightly irritating at the time, but should the fly have come from an infected source it may set up fatal pyæmia; several such cases are on record. Flies are probably also a potent source of epidemic diarrhœa, carrying the infective agent to the food supply of the child or directly to its mouth while asleep. The attacks of all the above insects, except the flea and the bug, can be guarded against by covering the face, while asleep or unwatched, with muslin. Similar agents may be used to keep away the insects as in the case of fleas.

Sulphur, internally, or applied externally in the form of soap, is supposed to keep all these insects away; so too is Chinosol in the bath. In the treatment of the bites, dabbing with Eau de Cologne and water will relieve the itching; or recourse may be had to vinegar and water, strong ammonia, carbolic acid 5 per cent., or lead lotion. A saturated solution of menthol in oil or spirit, about one part in four, can be applied as a spray or on cotton wool; or the bites may be painted over with ichthyol collodion. It cannot be too strongly impressed on nurses that in the case of babies the proper treatment is the prevention of the attacks of these parasites by careful watching, catching them when present, cleanliness of the child, nursery, and general surroundings; and that it ought never to be necessary to have recourse to the preventive and curative agents above mentioned, except as a temporary measure. They are, however, constantly needed for older children and when travelling, for many ships and beds are infested with either fleas or bugs. It is important to prevent the bites itching and thus to limit scratching and secondary infection.

